# Adenoid cystic carcinoma of the tongue – clinicopathological study and survival analysis

**DOI:** 10.1186/1758-3284-1-15

**Published:** 2009-05-29

**Authors:** Kuauhyama Luna-Ortiz, Tania Carmona-Luna, Ana María Cano-Valdez, Adalberto Mosqueda-Taylor, Angel Herrera-Gómez, Verónica Villavicencio-Valencia

**Affiliations:** 1 Departament of Head and Neck Surgery, Instituto Nacional de Cancerología, Av. San Fernando # 22 Col. Sección XVI, Tlalpan, México D.F. 14080, Mexico; 2 Universidad Nacional Autónoma de México (UNAM), Mexico; 3 Health Care Department, Universidad Autónoma Metropolitana Xochimilco, México; 4 Departament of Pathology, Instituto Nacional de Cancerología, Av. San Fernando # 22 Col. Sección XVI, Tlalpan, México D.F. 14080, Mexico

## Abstract

**Background:**

To review the demographic data of a series of adenoid cystic carcinoma (ACC) of the tongue, as well as to analyze c-kit expression, histopathologic patterns, prognostic factors, evolution, recurrences and/or persistence and survival.

**Methods:**

Retrospective study from 1986 to 2006, which reviews a database of 68 patients with diagnosis of head and neck ACC.

**Results:**

We found eight cases of ACC of the tongue (11.7% of all head and neck ACCs). There were 7 female (87.5%) and 1 male (12.5%) patients, with an average age of 51 years (range 33 to 67 years). Seven patients were surgically treated, three of which required adjuvant treatment. Only one female patient did not accept treatment. Average follow-up time was 5.3 years. Metastases developed in 37% of cases during the follow-up period. Histopathologically, the cribriform pattern predominated (6/8 cases). All cases presented perineural invasion, and one patient also presented vascular invasion. c-kit positivity was observed in all cases. Global survival in the seven treated cases was 51% and 34% at 5 and 10 years, respectively, while the disease-free period was of 64% at 3 years and 42% at 10 years.

**Conclusion:**

ACC of the tongue is a rare neoplasm, in which early diagnosis is important because these are slowly-growing tumors that produce diffuse invasion. As the role of c-kit could not be assesed in this series, surgery continues to be the cornerstone of treatment and radiotherapy is indicated when surgical margins are compromised. Metastatic disease is still hard to handle because of the lack of adequate therapies for these tumors. Hence, survival has not changed in the last years.

## Introduction

Adenoid cystic carcinoma (ACC) is a malignant neoplasm that originates in both the minor and major salivary glands, characterized by slow growth, diffuse invasion and potential to produce distant metastases, mainly to the lungs and bones [1]. It is an infrequent lesion, as it represents approximately 1% to 2% of all malignant neoplasms of the head and neck, and up to 10% to 15% of all malignant salivary gland neoplasms [2]. The most common intraoral site for minor salivary gland tumors is the hard palate, followed by the base of the tongue [3] where up to 96% of all tumors are malignant, and ACC represents 30% of them [4,5]. On the other hand, one of the least frequent sites of presentation for ACC is the mobile tongue, as several authors have reported an incidence of only approximately 3% of the cases [6]. Likewise, it has been reported that in this location, 75% are in stages T3 and T4 at the time of diagnosis [7]. Histopathologically, ACC may present in three different patterns: solid, tubular, and cribriform; perineural invasion is common and has been reported in almost half of the cases. Necrosis and vascular invasion are present at a lower rate [6].

The cornerstone of treatment is surgery, while radiotherapy has been considered for advanced T stages and as adjuvant in the presence of positive microscopic margins [8]. Some authors suggest that advanced and non-resectable tumors may be treated only with radiotherapy [9].

It has recently been found that c-kit (CD-117), a tyrosine-kinase receptor involved in growth and development of normal tissues and in some neoplasms [10] expresses in ACC; previous studies have found its expression in 78% of 45 cases[11] and in 78.5% of 14 cases[10], which led some to consider the use of tyrosine kinase inhibitors such as imatinib mesylate as an adjuvant and/or therapeutic tool to manage distant metastases[12].

The objectives of our study were to review the demographic data of ACC of the tongue diagnosed at our institution, to assess c-kit expression in these tumors and to describe the histopathological patterns, prognostic factors (such as perineural and vascular invasion), as well as evolution, recurrences and/or persistence and survival of patients.

## Methods

This is a retrospective study that comprises the period of 1986 to 2006, which reviews a database of 68 patients with ACC of the head and neck region treated at the Instituto Nacional de Cancerologia of Mexico. We included in this study only those patients with ACC of the tongue. Their clinical stage at the time of admission was determined according to the AJCC 2002[13]. We reviewed the clinical files and performed a new histopathological study of each patient to assess stage and microscopic pattern, perineural and vascular invasion and c-kit (CD 117) expression, except in one patient for which only the slides but not the paraffin blocks were available. Demographic and clinical information were collected from the clinical files, including the following data: age, gender, time of evolution prior to diagnosis, TNM classification, initial and adjuvant treatment, presence of perineural invasion, recurrences, presence of metastases, general overall survival and disease-free survival at 5 and 10 years according to Kaplan and Meier.

## Results

We found eight cases of ACC of the tongue (6 at the base and 2 in the mobile tongue), which represent 11.8% of all ACC of the head and neck area assessed at our institution during the period of study. There were seven female (87.5%) and one male (12.5%) patients with an average age of 51 years (range 33–67 years). All cases were staged, except for one recurring patient. The salient clinical features are shown in Table 1.

**Table 1:** Clinical features and treatment of ACC.

Case	Gender	Age	Location	T	N	M	CS	Surgery performed
1	F	38	Base of tongue (left)	4	0	0	IVA	Left hemiglossectomy

2	F	48	Base of tongue (left)	-	-	-	No*	Total glossectomy + total laryngectomy + floor of mouth resection + reconstruction with pectoral flap

3	F	64	Base of tongue (left)	2	1	0	III	Hemiglossectomy + left base of tongue excision + supraomohyoid left neck dissection + reconstruction with pectoral flap.

4	F	40	Base of tongue (left)	4	0	X	IVA	Total glossectomy + floor of mouth resection + reconstruction with pectoral flap.

5	F	33	Mobile tongue	4	2c	1	IV	Total glossectomy + modified right neck dissection + supraomohyoid left neck dissection + reconstruction with microvascular flap from the anterior abdominal rectus abdomen.

6	F	57	Mobile tongue	3	1	0	III	Marginal resection with CO_2 _laser + antero-lateral dissection of the left neck

7	M	67	Base of tongue (right)	3	0	0	III	Primary tumour resection + radical right neck dissection.

8	F	63	Base of tongue	3	0	0	III	Did not accept treatment

*not classified for being a recurrence. CS: clinical stage

Seven patients were treated with surgery, three of which required adjuvant treatment: two with radiotherapy (average of 59 Gy per patient), one due to perineural invasion and the other because of presenting regional lymph node metastases. The latter patient received concomitant chemo-radiotherapy due to positive surgical margins aside from the regional metastases. Only one female patient of the original eight did not accept treatment. The average follow-up time was 5.3 years. During follow-up, 37% of the patients presented pulmonary metastases (one accompanied by bone metastases) (Table 2). Histopathologically, the cribriform pattern predominated (6/8 cases). All patients presented perineural invasion, and one case presented also vascular invasion. Immunohistochemistry with c-kit was positive in all the reviewed cases (Table 3).

**Table 2:** Follow-up of treated patients.

Case	Metastatic lymph nodes	Distant metastases	Follow-up time **(months)**	Current *Status*
1	No	No	207	AWoD

2	No	Bilateral pulmonary	84	DWD

3	Yes, lymph node conglomerate	No	84	LWoD

4	No	Bilateral pulmonary	60	DWD

5	2/8 base of tongue	Pulmonary and bone	2.5	LWD

6	1/38	No	6	AWoD

7	2/39	No	0	DWoD

LWoD: Lost without disease, LWD: lost with disease, DWD: dead with disease, AWoD: alive without disease, DWoD: dead without disease.

**Table 3:** Anatomopathologic assessment of ACC of tongue.

Case	Stage	Pattern	Perineural invasion	Vascular invasion	Margins	c-kit expression
1	II	Cribriform	yes	No	-	+

2	II	Cribriform	yes	No	-	+

3	II	Cribriform	yes	No	-	+

4	I	Tubular	yes	No	-	+

5	III	Solid	yes	Yes	-	+

6	II	Cribriform	yes	No	+	+

7	II	Cribriform	yes	No	-	+

8	II	Cribriform	yes	No	*	+

* Patient did not accept surgery

Overall survival in the seven treated cases was 51% and 34% at 5 and 10 years, respectively. Disease-free survival was 64% at 3 years and 42% at 5 and 10 years.

## Discussion

Neoplasms of accesory salivary gland origin occur less commonly than those arising from major salivary glands. Several studies indicate that malignant lesions account for a greater percentage of accesory salivary gland tumors as compared to major salivary glands. Tongue is a relatively uncommon site for salivary gland neoplasms [14].

ACC is an extremely unpredictable neoplasm regarding its evolution and behavior as it grows slowly, regional metastases are infrequently seen, but it tend to produce late distant metastases; however this condition does not lead to a short term death. In the same way as other authors who report ACC series of the head and neck [4,8,15,16], we also found that ACC located in the tongue occurs more frequently in female patients.

Previous series of ACC of the head and neck region have shown that the frequency in wich the tongue has been the site of origin ranged from 3.4% to 17.1% [3,4,11,15,17]. We found a similar figure in our series of 68 cases, where tongue location represented 11.8%, which is also similar to Khafif et al. [3] results. Also similar was the fact that in that study mobile tongue was the primary location in 2.9% and the base of the tongue in 8.8%, whereas we found a 3% and 8.8% respectively for such locations.

**Figure 1:**
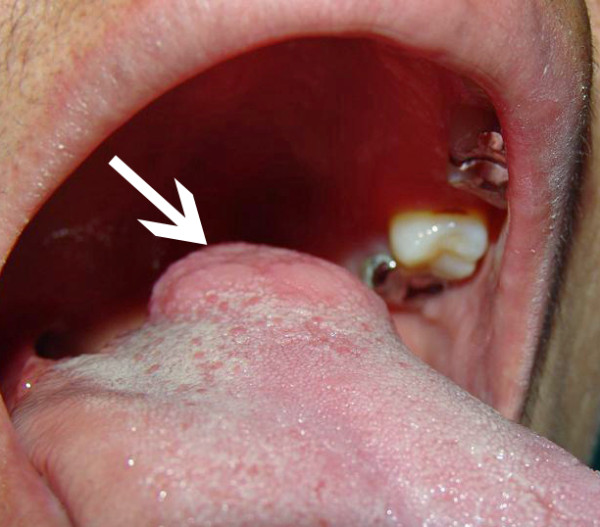
**57 year old female with a lateral mobile tongue ACC**.

**Figure 2:**
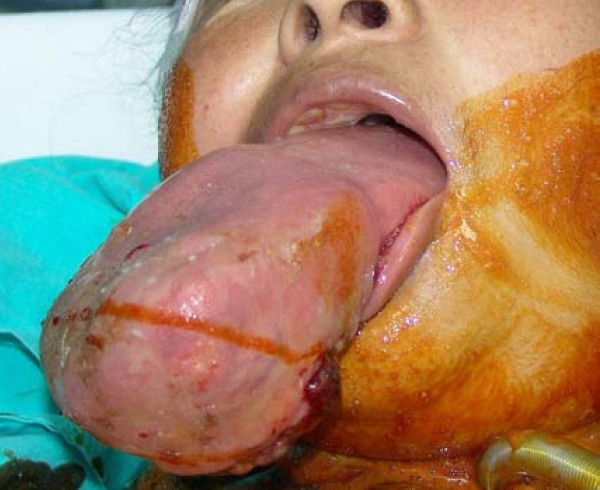
**33 year old female with a massive infiltration of the tongue due to ACC**.

**Figure 3:**
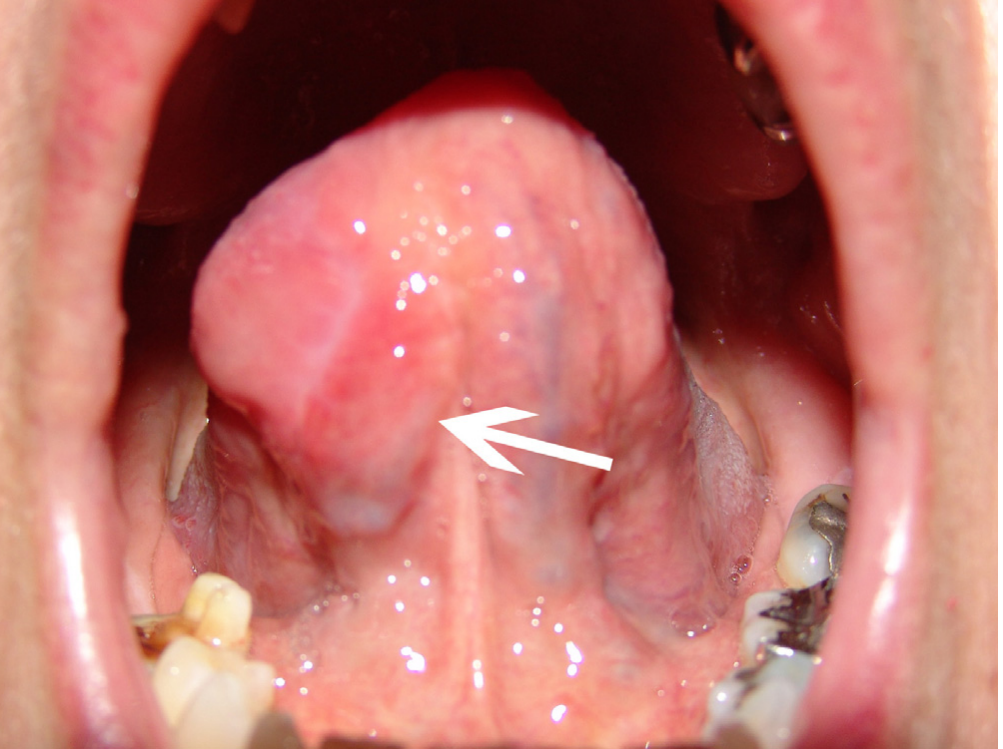
**An anterolateral tumor of the mobile tongue shown by the arrow**.

With respect to head and neck cases of ACC, Silverman et al. [8] found that 46.3% were in clinical stages I and II and 53.7% in stages III and IV; in contrast, the report of 34 patients by Khafif et al. [3] showed 64% in early stages (I and II), and 35% in advanced stages (III and IV). In our study we had 87.5% of cases in advanced clinical stage, which is higher than what has been reported in the literature. These diferences may be explained by the fact that when located in the tongue, the clinical course of this neoplasm is usually asymptomatic, with gradual submucosal tumoral growth, which hinders an early diagnosis; consequently the time elapsed from the first clinical manifestation through diagnosis is long, as it has been estimated from 6 months to 8 years[16], while in our series the mean was 15 months. In this sense, most tumors located at the base of the tongue are in advanced stages at the time of diagnosis, as shown by Goepfert et al[7], who found that 75% of their ACC cases were in stages T3 and T4, which is similar to our findings. On the other hand, although tumors located in the mobile tongue produce more functional alterations and hence, should be diagnosed at an earlier stage, this was not seen in our series, where tumors ranged in size from 2 to 15 cm (Figures 1, 2, 3). The reason of this is not clear, but it may be related to the lack of pain in several cases, as well as socio-cultural and economic background of our patients.

Surgery is the cornerstone of treatment, and its extension depends primarily on the size of the tumor. It may be performed in the form of partial glossectomy, hemiglossectomy, total glossectomy with/without preservation of the larynx, or total glossectomy with/without reconstruction if the case merits it. For cases requiring reconstruction, this can be accomplished with a flap, either pedunculated (from the pectoral) or free microvascular flap (wide dorsal muscle, abdominal rectus, antebranchial) [18,19]. The relevance and extent of surgery relies on the early diagnosis and, consequently, on the T stage. As can be observed in our series dealing with advanced stages, total glossectomy and larynx preservation had to be performed in four cases with pectoral flap reconstruction in three of them, and in one case with a free flap from the abdominal rectus; the latter, in our experience, is the best option as it provides adequate volume [20].

The use of adjuvant radiotherapy is still controversial; on one side, there are authors that recommend it for an advanced T stage with positive margins [8], whereas other authors have obtained an increase in local control rate at 5 or 10 years, advocating for this therapeutic option in all patients [9]. We used radiotherapy in three cases. Based in our experience with other head and neck tumors [21], we believe that concomitant chemo-radiotherapy represents an alternative for patients with advanced disease or to preserve organs, but its use has not been explored as primary treatment or as post-surgical adjuvancy. Besides, as it is well known, it is relatively inefficacious in the treatment of ACC, perhaps due to the slow growth rate of this neoplasm. In this respect, several agents have been proven with low rates of response, such as cisplatin, 5-fluorouracil, adriamycin and cytoxan, either alone or in combination [22].

Relevance of c-kit expression lies in its possible role to direct treatment of non-resectable or metastatic disease, although the use of imatinib mesylate as tyrosine kinase inhibitor is still controversial. In this respect, there are some contradictory results in the literature, as for example, favorable results have been presented by Alcedo et al. [12] in 2 cases of unresectable ACC treated with imatinib mesylate, one for recurrent disease and the other for a locally advanced tumor; however there are other authors who have observed progression of the disease during treatment with imatinib mesylate[23]. The seven patients in whom c-kit expression was assessed were all positive; however, c-kit activation was not assessed, which would be an important step to decide to treat or not with tyrosine kinase inhibitors, because mutations in 11 c-kit and 9 c-kit exons condition an 83.5% and 47.8% response, respectively, in contrast to patients with non detectable c-kit mutation, who do not present an objective response [24]. As the results at present are derived from small series or from isolated case-reports with more questions than answers on the benefit of tyrosine kinase inhibitors and due to their high cost, we have not been able to pursue treatment with these drugs in our patients.

In our study, one case presented distant metastases at the time of diagnosis and two during the follow-up (2 pulmonary and 1 pulmonary and bone metastases). These patients have had a follow-up of 7 years, 5 years, and 2.5 months, respectively, and have died due to causes related to the neoplasm. One female patient presented a regional lymph node conglomerate, which was treated with neck dissection and adjuvant radiotherapy; after 7 years of follow-up she is currently without data of tumoral activity. These findings suggest that even in spite of advanced staged or metastatic disease it is possible to offer radical surgical treatment, as these patients may follow a slow course of the disease with longer survival as compared to other types of tumors with similar advanced disease.

The most common histological subtype in our study was the cribriform, just like in other series. Its relevance lies in that the solid subtype depicts the worst prognosis, with a survival of 34% at 10 years, in contrast to the 76% of the cribriform and the 100% of the tubular subtypes [25]. The histological subtypes of lower-grade malignancy (tubular and cribriform) have a better prognosis than those of high malignancy (solid) as the latter are associated with recurrences, early distant metastases, and a higher mortality rate [25], as occurred in our female patient (No 5), the only one with solid pattern, who presented the largest tumor (15 × 11 cm) in the mobile tongue, with a growth evolution of only 10 months. The presence of perineural invasion was found in 100% of our cases. Previous studies have shown that this implies a worse prognosis, decreasing survival to 76% against 100% of those not presenting it [25]. According to the AFIP (Armed Forces Institute of Pathology) the tongue is the site of occurrence of malignanat neoplasms in 58.2% in males and 56.6% in females, and ACC predominates in the 5^th ^and 6^th ^decade of life. From a registry of all ACC (600 cases) tongue represented a 17.1% with 30 cases [17].

In this study, overall survival was similar to that reported in other series of ACC of the head and neck, as that of da Cruz Perez et al. [5] from Brazil, who showed 56.5% and 32.5% at 5 and 10 years respectively; this contrasts with the disease-free survival, which in our cases was 42% at 10 years, and only 29% in the previously reported Brazilian study[5], which could be related to more radical treatment modalities employed in our cases.

## Conclusion

ACC of the tongue is a very infrequent neoplasm; early diagnosis and proper treatment are important factors from a functional point of view, since these are slowly-growing tumors that may produce diffuse invasion. As the role of c-kit could not be assesed in this series, surgery continues to be the cornerstone of treatment and radiotherapy is indicated in the presence of compromised surgical margins. Metastatic disease is still hard to handle because of the lack of adequate therapies for these tumors and therefore survival has not changed in the last years.

## Competing interests

The authors declare that they have no competing interests.

## Authors' contributions

KLO conceived and desing of the study and write the paper and as a surgeon of the cases. TCL participated in the dising of the study, search the data base an analysis of them. AMaCV reviewed the pathology and write pathological discussion. AMT reviewed the pathology and write pathological discussion, and reviewed of the article when the paper was ended. AHG Analysis of the data base, reviewed of the papar and was one of the surgeons. VVV participated in the dising of the study and performed the statistical analysis. All authors read and approved the final manuscript.
